# Reference intervals for hemoglobin and mean corpuscular volume in an ethnically diverse community sample of Canadian children 2 to 36 months

**DOI:** 10.1186/s12887-021-02709-w

**Published:** 2021-05-19

**Authors:** Jemila S. Hamid, Eshetu G. Atenafu, Cornelia M. Borkhoff, Catherine S. Birken, Jonathon L. Maguire, Mary Kathryn Bohn, Khosrow Adeli, Mohamed Abdelhaleem, Patricia C. Parkin

**Affiliations:** 1 Department of Mathematics and Statistics, University of Ottawa, Ottawa, Canada; 2 Biostatistics Department, Princess Margaret Cancer Center, University Health Network, Toronto, Canada; 3 Pediatric Outcomes Research Team (PORT), Division of Pediatric Medicine and Sick Kids Research Institute, Hospital for Sick Children, Toronto, Canada; 4 Institute of Health Policy, Management and Evaluation, University of Toronto, Toronto, Canada; 5 Department of Pediatrics, Faculty of Medicine, University of Toronto, Toronto, Ontario Canada; 6 Department of Pediatrics, and Li Ka Shing Knowledge Institute, St. Michael’s Hospital, Toronto, Canada; 7 CALIPER Program, Pediatric Laboratory Medicine, The Hospital for Sick Children, Toronto, Canada; 8 Department of Laboratory Medicine & Pathobiology, Faculty of Medicine, University of Toronto, Toronto, Canada; 9 Haematopathology, Pediatric Laboratory Medicine, The Hospital for Sick Children, Toronto, Canada; 10 The Hospital for Sick Children Research Institute, Peter Gilgan Centre for Research and Learning, 686 Bay St, Toronto, ON M5G 0A4 Canada

**Keywords:** Reference intervals, Hemoglobin, Mean corpuscular volume

## Abstract

**Objective:**

To establish reference intervals for hemoglobin and mean corpuscular volume (MCV) in an ethnically diverse community sample of Canadian children 36 months and younger.

**Methods:**

We collected blood samples from young children at scheduled primary care health supervision visits at 2 weeks, 2, 4, 6, 9, 12, 15, 18, 24, and 36 months of age. Samples were analyzed on the Sysmex XN-9000 Hematology Analyzer. We followed the Clinical and Laboratory Standards Institute guidelines in our analysis. Data were partitioned by sex and also combined. We considered large age partitions (3 and 6 months) as well as monthly partitions. Reference intervals (lower and upper limits) and 90% confidence intervals were calculated.

**Results:**

Data from 2106 children were included. The age range was 2 weeks to 36 months, 46% were female, 48% were European and 23% were of mixed ethnicity. For hemoglobin, from 2 to 36 months of age, we found a wide reference interval and the 90% confidence intervals indicated little difference across age groups or according to sex. For MCV, from 2 to 7 months of age there was considerable decrease in the reference interval, which was lowest during the second year of life, followed by a slight increase in the last months of the third year of life.

**Conclusion:**

These findings suggest adoption of a single hemoglobin reference interval for children 2–36 months of age. Further studies in children under 4 months of age are needed.

**Trial registration:**

*TARGet Kids!* cohort is registered at ClinicalTrials.gov. www.clinicaltrials.gov. Identifier: NCT01869530.

**Supplementary Information:**

The online version contains supplementary material available at 10.1186/s12887-021-02709-w.

## Introduction

Hemoglobin and mean corpuscular volume (MCV) are commonly ordered laboratory tests in infants and young children, especially for the assessment of common conditions such as iron deficiency which peaks in prevalence in this age group [1]. Reference intervals are derived from apparently healthy populations and presented as two limits (lower and upper limit). The lower and upper limits of reference intervals correspond to the 2.5th and 97.5th percentiles of the distribution of a healthy population, and hence provide an interval in which 95% of the population lies. Although reference intervals do not define the presence or absence of disease states, they play an important role in the interpretation of laboratory tests and commonly accompany test results. Despite being commonly used, there is little data to support pediatric hematologic reference intervals, in particular for young children [2]. Animal and human studies suggest that the capacity for iron homeostasis develops during infancy and early childhood [3, 4]. Therefore, it is important to establish age-specific reference intervals for hematologic parameters for this age group [2].

Developing reference intervals for young children is challenging for several reasons. First, it is difficult to obtain blood from a large enough sample of young, healthy children [2, 5]. Thus, some reference intervals have been established using blood samples from children cared for in inpatient, outpatient and emergency department settings and who may have acute or chronic illness [6, 7]. Second, samples of fresh whole blood are required to measure hematologic parameters. The time-sensitive nature of this blood collection poses another difficulty [2].

The Clinical and Laboratory Standards Institute (CLSI) provides guidelines for establishing reference intervals including selection of subjects, sample size requirements, approaches to the detection and removal of outliers, and approaches to creating partitions (groupings) for age and sex [8]. A systematic review of pediatric reference intervals published in 2014 reported that only one third of the included studies included (31.81%, 7 of 22) followed the CLSI guidelines [9]. Following that, a methodology paper was published, where extensive simulations and empirical evaluations were performed to show optimality of the various statistical methods with respect to distribution of analytes, heterogeneity involved in the measurements as well as sample size [10]. In recent years there have been improvements in adherence to the CLSI guidelines and optimal use of statistical methods in establishing reference intervals [11]. In this study, we followed the CLSI guideline as well as the strategies outlined in the methodology paper in choosing optimal statistical methods for estimating reference limits for each of the partitions.

Hematologic reference intervals have been developed in healthy Canadian children over 3 years of age [2, 12]. However, there are few publications establishing reference intervals for hemoglobin and mean corpuscular volume (MCV) in young children under 3 years using CLSI guidelines. Two publications report reference intervals using CLSI guidelines for young children from China and Korea, but neither included children under 4 months of age [13, 14].

The objective of our study was to establish reference intervals for hemoglobin and mean corpuscular volume (MCV) in an ethnically diverse community sample of Canadian children 36 months and younger using the Clinical and Laboratory Standards Institute guidelines.

## Methods

### Study design

We used a cross-sectional study design.

### Study population

We created a primary care practice-based research network called *TARGet Kids!* in Toronto, Canada in 2008 (www.targetkids.ca). The research network utilizes the opportunity of government-funded health supervision visits to obtain rich clinical data including blood samples from consenting participants. These visits are scheduled at 2 weeks, 2, 4, 6, 9, 12, 15, 18, 24, and 36 months of age; however, these are approximations as many children are unable to visit at exactly these ages. Research assistants trained in pediatric phlebotomy are embedded in each primary care practice and recruit healthy young children into the *TARGet Kids!* cohort during a scheduled health supervision visit. Exclusion criteria for the cohort are children with: known health conditions affecting growth, chronic conditions (other than asthma and autism), severe developmental delay, gestational age less than 32 weeks, visit for an acute illness, and families unable to communicate in English. Parents provide informed consent and complete a questionnaire, including the child’s age and sex, and maternal and paternal ethnicity. The cohort profile has been previously described [15]. This ongoing longitudinal cohort has been approved by the Research Ethics Boards at the Hospital for Sick Children and St. Michael’s Hospital, Toronto, Canada, and registered at Clinicaltrials.gov (NCT01869530).

For the current study, we used data collected on children from 2 weeks to 36 months of age. If a child had a blood sample collected at more than one visit, the sample from the first visit was used.

### Sample collection and analysis

The research assistants collected blood samples in lavender EDTA tubes. Fresh blood samples were transported to the laboratory at Mount Sinai Services, Inc. the same day (http://www.mountsinaiservices.com/). Blood samples were analyzed within 4 to 6 h of collection. Samples were analyzed on the Sysmex XN-9000 Hematology Analyzer (Japan). Data from 2106 children were included in our analysis.

### Statistical analysis

We followed the strategies provided in CLSI C28-A3 guidelines in establishing our reference intervals. Moreover, additional optimality criteria from a published methodology paper were also utilized for selecting appropriate statistical methods [8, 9]. Data were initially partitioned by sex (female/male) and clinically meaningful age categories [8]. The trend of each of the analytes was explored and visually analyzed through scatter plots to determine appropriate age partitions for both females and males. Age partitions were then created based on the observed trend, clinical knowledge of child growth and development, and current knowledge regarding these analytes. Once appropriate partitions were created, outliers were identified and removed from each partition using outlier detection techniques described by Horn et al. [16]

For each partition, distributions of the analytes were re-examined through histograms, normal probability plots and by using the Shapiro-Wilks test of normality [17]. Skewness parameters were also estimated and evaluated to detect the extent of deviation from the Gaussian distribution. The lower and upper limits of the reference intervals corresponding to the 2.5th and 97.5th percentiles of the distributions were then estimated for each partition. Appropriate statistical methods were then selected based on sample size, result of the test of normality and extent of the skewness of the distributions. We selected the parametric, non-parametric or robust methods according to the optimality criteria provided in a publication consisting of extensive simulations [10]. We created heatmaps showing the methods we used for each of the partitions along with the parameter estimates of the sampling distributions. The 90% confidence intervals (CIs) were provided for the lower and upper limits of all reference intervals. Difference between partitions were examined using a t-test, where we compared means between adjacent partitions. Statistical significance was determined at α = 0.05. All analyses were performed using the R Statistical Software version 3.2.3 [18].

Once the above initial analyses were performed, we also considered reference intervals for smaller age intervals, when adequate sample size was available. The basis for conducting such analyses was: 1) the observed continuous trend for the analyte values across age; and 2) previous knowledge, where the analyte values were believed to decline in the months after birth, then increase and stabilize thereafter. First, we used monthly age partitions, when sample size was sufficient, to properly capture the somewhat continuous trajectory across age for children 36 months and younger. We then created quarterly partitions, to provide as narrow an age partitioning as possible for those instances where we had limited sample size for monthly partitions. In the analysis involving these narrow age partitions, the data for females and males were combined after confirming that there were no statistical differences between the partitions corresponding to the two sexes. This allowed us to maximize sample size and provide more accurate estimates and more precise confidence intervals for the upper and lower reference limits. Quarterly and monthly age partitions were considered for all partitions with a sample size of 20 or above. Through simulation, we have shown that 20 is the sample size under which optimality of estimates (bias and precision) is very poor [10].

## Results

Of the total 2106 children, 968 (46%) were female and 1138 (54%) male. Age ranged from 2 weeks to 36 months. Participant characteristics are shown in Table 1. Child ethnicity was European (Western and Eastern) for 1013 (48.1%); for the remainder, ethnicity was diverse, including 479 (22.7%) of mixed ethnicity.

**Table 1:** Participant characteristics

Characteristic	
**Age in months**: median (IQR^a^)	15 (12, 2)
**Sex:** n (%)	
Female	968 (46.0)
Male	1138 (54.0)
**Ethnicity:** n (%)	
**Maternal**	
European	1214 (57.6)
East Asian	106 (5.0)
South Asian	160 (7.6)
Southeast Asian	66 (3.1)
Arab	40 (1.9)
African	107 (5.1)
Latin	58 (2.8)
Mixed	125 (5.9)
Other	8 (0.4)
Missing	222 (10.5)
**Paternal**	
European	1223 (58.1)
East Asian	66 (3.1)
South Asian	162 (7.7)
Southeast Asian	53 (2.5)
Arab	47 (2.2)
African	142 (6.7)
Latin	50 (2.7)
Mixed	115 (5.5)
Other	9 (0.4)
Missing	239 (11.4)
**Child**	
European	1013 (48.1)
East Asian	42 (2.0)
South Asian	129 (6.1)
Southeast Asian	39 (1.9)
Arab	26 (1.2)
African	80 (3.8)
Latin	28 (1.3)
Mixed^b^	479 (22.7)
Other	2 (0.1)
Missing^c^	268 (12.7)

^a^*IQR* Inter Quartile Range

^b^A child is considered of mixed ethnicity if 1) parents are of different ethnicity 2) at least one of the parents is mixed

^c^A child’s ethnicity is categorized as missing if neither of the parents are of mixed ethnicity and the ethnicity of at least one of the parents is missing

**Fig. 1:**
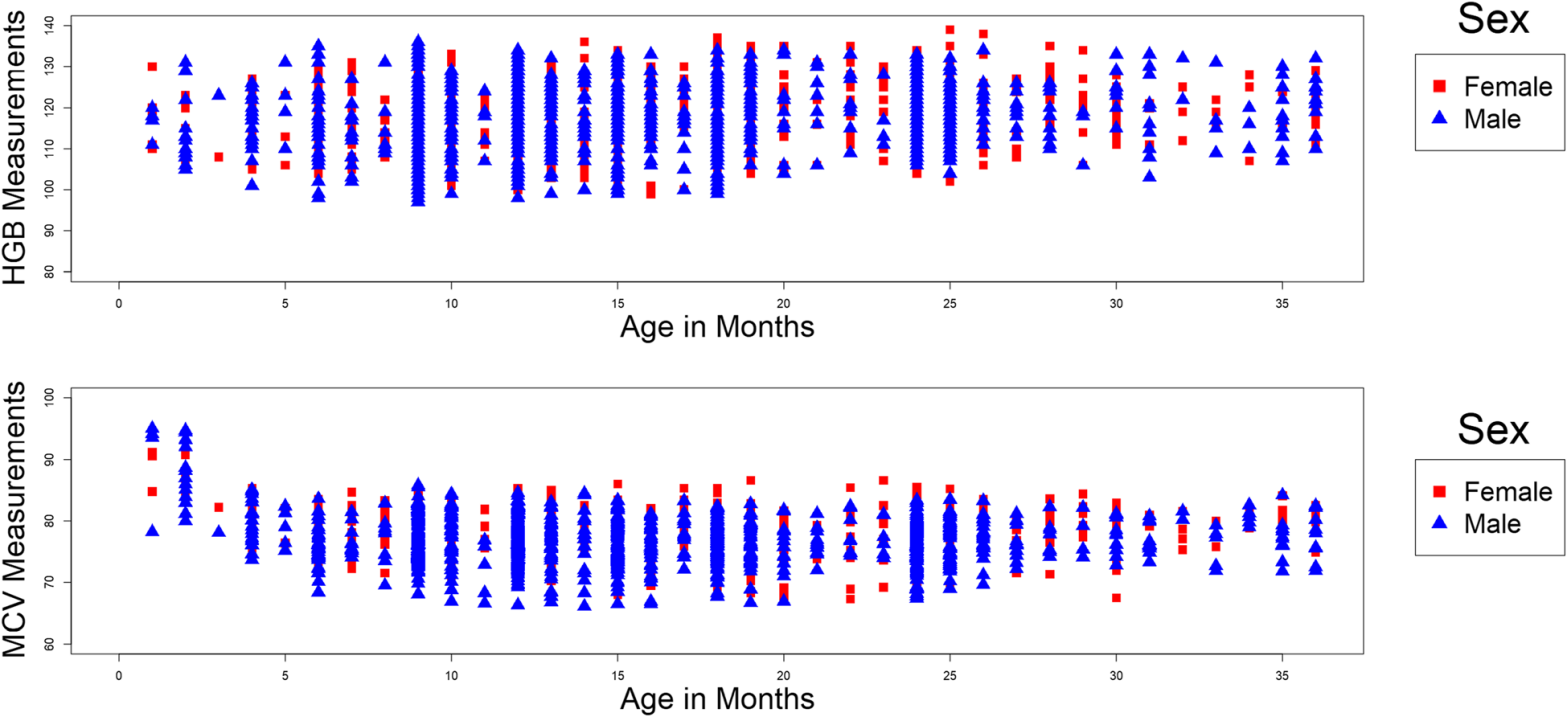
**a** and **b** Age-specific scatter plots by sex for hemoglobin (**a**) and MCV (**b**) for children from 2 weeks to 36 months of age

Age-specific scatter plots by sex for hemoglobin and MCV measurements are provided in Fig. 1a and b. As seen in the Figures, for children under the age of 3 years, measurements for the analytes did not differ with respect to sex. Nonetheless, we provided sex-specific reference intervals following common practice as well as for comparison purposes.

Observations from Fig. 1a and b were used in creating the age partitions. The Figures showed no trend with respect to age for hemoglobin, except a slight decreasing trend under the age of 5 to 6 months. For MCV, an overall declining trend was observed, with apparent stabilization around 6 to 7 months of age.

We initially considered a total of 18 partitions (with respect to sex and age) and the reference interval estimates along with 90% CIs are provided in Table 2. The skewness estimates for each partition with corresponding *p*-values for the test of normality are provided in the Supplementary material (Table A1). A heatmap representing the methods of reference interval estimation used for each of the partitions is also provided in the Supplementary material (Table A2). Considering that no difference in analytes was observed between females and males (Fig. 1a and b), we combined data to maximize sample size and provided reference intervals using the combined population, hence allowing more accurate and precise estimators to be obtained (Table 3). The sample size, skewness estimates and *p*-values corresponding to these combined reference intervals are provided in the Supplementary material (Table A3). The corresponding heatmap showing which statistical method was used is provided in the Supplementary material (Table A4).

**Table 2:** Hemoglobin and mean capsular volume reference intervals (90% confidence intervals)

Age Group	Reference Intervals (90% CI)					
	Female			Male		
	n	Lower	Upper	n	Lower	Upper
**Hemoglobin (HGB)**						
**Year 1**	**360**	**102.0 (100.4–103.0)**	**130.8 (129.7–131.9)**	**425**	**100.6 (99.5101.8)**	**132.7 (131.6–133.8)**
Month 1 – Month 6	62	102.0 (99.2–105.0)	130.8 (128.2–133.5)	82	100.7 (98.2–103.1)	131.2 (128.8–133.6)
Month 7 – Month 12	298	101.0 (100.2–103.0)	130.8 (129.6–132.0)	343	100.6 (99.4–101.9)	133.1 (131.8–134.3)
**Year 2**	**441**	**104.0 (102.6–105.0)**	**134.3 (133.3–135.4)**	**503**	**103.0 (100.0–105.0)**	**132.0 (131.0–133.0)**
Month 13 – Month 18	248	103.0 (101.1–104.0)	134.4 (133–135.9)	292	102.3 (101.0–103.6)	132.0 (131.0–133.0)
Month 19 – Month 24	193	105.0 (103.7–107.0)	134.1 (132.6–135.6)	211	105.5 (104.5–106.8)	132.5 (131.2–133.9)
**Year 3**	**129**	**105.0 (103.6–107.0)**	**133.5 (131.7–135.2)**	**154**	**106.1 (104.5–107.6)**	**132.8 (131.2–134.3)**
Month 25 – Month 30	101	105.0 (102.7–107.0)	134.5 (132.4–136.6)	113	107.3 (105.6–109.0)	132.1 (130.5–133.8)
Month 31 – Month 36	28	108.0 (105.0–111.0)	129.3 (126.4–132.3)	41	103.4 (99.9–106.9)	134.4 (130.9–137.9)
**Mean Capsular Volume (MCV)**						
**Year 1**	**356**	**72.2 (71.5–72.9)**	85.3 (84.5–87.9)	**428**	**69.7 (68.4–70.1)**	**87.0 (84.9–93.2)**
Month 1 – Month 6	60	71.2 (69.5–72.8)	89.0 (87.3–90.6)	82	70.1 (70.1–70.1)	94.7 (94.7–94.7)
Month 7 – Month 12	296	72.0 (71.5–73)	84.2 (83.3–84.8)	346	70.0 (69.5–70.6)	84.0 (83.0–84.6)
**Year 2**	**435**	**69.0 (68.5–70.0)**	**84.6 (83.4–85.3)**	**504**	**67.8 (66.9–68.9)**	**82.4 (82.2–83.1)**
Month 13 – Month 18	246	71.0 (70.6–72.0)	84.6 (84.0–85.2)	292	67.7 (66.5–68.5)	82.5 (82.2–83.4)
Month 19 – Month 24	189	69.0 (67.7–70.0)	84.3 (83.4–86.6)	212	67.8 (66.9–69.1)	82.4 (81.7–83.0)
**Year 3**	**126**	**72.0 (71.1–73.0)**	**84.1 (82.8–85.2)**	**154**	**70.9 (70.2–71.6)**	**83.4 (82.7–84.2)**
Month 25 – Month 30	100	71.0 (70.4–72.0)	84.7 (83.7–85.7)	112	70.7 (69.9–71.6)	83.2 (82.3–84.0)
Month 31 – Month 36	26	75.0 (73.4–76.0)	84.2 (82.8–85.5)	42	71.4 (70.0–72.8)	84.0 (82.6–85.4)

**Table 3:** Reference intervals (90% CI) for combined female/male for hemoglobin and mean capsular volume

Age	Reference Intervals (90% CI)	
	Lower	Upper
**Hemoglobin (HGB)**		
**Year 1**	**101.0 (100.2–101.8)**	**131.8 (131.1–132.6)**
Month 1 – Month 6	101.2 (99.4–103.0)	131.0 (129.2–132.8)
Month 7 – Month 9	100.0 (98.7–101.4)	130.8 (129.4–132.2)
Month 10- Month 12	101.7 (100.6–102.9)	132.7 (131.6–133.8)
**Year 2**	**103.6 (102.9–104.3)**	**133.7 (133.3–135.4)**
Month 13 – Month 15	102.5 (101.2–103.9)	132.0 (130.0–133.0)
Month 16 – Month 18	102.3 (100.9–103.7)	134.1 (132.8–135.5)
Month 19 – Month 21	104.8 (103.2–106.7)	134.9 (133.1–136.8)
Month 22 – Month 24	105.7 (104.5–106.9)	132.4 (131.3–133.6)
**Year 3**	**105.9 (104.7–107)**	**133.1 (131.9–134.2)**
Month 25 – Month 30	106.1 (104.8–107.4)	133.4 (132.1–134.6)
Month 31 – Month 36	105.1 (102.8–107.5)	132.7 (130.1–135.0)
**Mean Capsular Volume (MCV)**		
**Year 1**	**70.7 (69.8–71.5)**	85.8 (84.8–88.2)
Month 1 – Month 6	68.9 (67.6–70.2)	90.9 (89.6–92.3)
Month 7 – Month 9	71.2 (70.6–71.8)	84.4 (83.8–85.0)
Month 10- Month 12	70.1 (68.8–71.3	84.3 (83.8–84.7)
**Year 2**	**68.5 (68–69.1)**	**83.4 (82.9–83.7)**
Month 13 – Month 15	68.4 (66.8–70.1)	83.9 (83.2–85.0)
Month 16 – Month 18	68.9 (67.7–70.1)	82.9 (82.3–84.6)
Month 19 – Month 21	69.6 (68.7–70.4)	82.8 (81.7–86.6)
Month 22 – Month 24	68.9 (67.4–69.2)	83.6 (82.4–85.4)
**Year 3**	**71.3 (69.2–71.9)**	**83.5 (82.5–84.2)**
Month 25 – Month 30	70.2 (69–71.8)	83.5 (82.5–84.4)
Month 31 – Month 36	72.4 (71.5–73.9)	84.4 (83.3–85.4)

We calculated reference intervals for quarterly (3 month period) and monthly partitions, with data from females and males combined. The results are provided in Table 4. Sample sizes, skewness estimates as well as *p*-values for normality corresponding to partitions in Table 4 are provided in the Supplementary material (Table A5), and the heatmap showing the statistical methods used is also provided in the Supplementary material (Table A6). The number of children in a monthly partition for females and males separately are provided in the Supplementary material (Table A7).

**Table 4:** Monthly and quarterly reference intervals for hemoglobin and mean capsular volume

Age	Reference Intervals [90% CI]			
	Hemoglobin		Mean Capsular Volume (MCV)	
	Lower	Upper	Lower	Upper
**Year 1**				
**First Quarter**	**94.5 (90.1–98.9)**	**130.0 (125.6–134.4)**	**78.1 (75.4–80.7)**	**99.5 (96.8–102.2)**
Month 1	–	–	–	–
Month 2	101.0 (100.2–103.0)	127.7 (122.1–133.3)	79.1 (76.5–81.6)	95.5 (93.0–98.1)
Month 3	–	–	–	–
**Second Quarter**	**102.6 (100.7-104.5)**	**130.5 (128.6–132.4)**	**70.6 (69.6–71.6)**	**85.1 (84.1–86.1)**
Month 4	102.4 (98.9-106.0)	129.3 (125.7–132.8)	73.4 (71.6–75.1)	86.9 (85.1–88.6)
Month 5	–	–	–	–
Month 6	102.8 (100.4-105.2)	130.9 (128.5–133.3)	70.0 (68.9–71.2)	83.5 (82.4–84.7)
**Third Quarter**	**100.4 (99.1-101.7)**	**129.8 (128.5–131.2)**	**71.0 (70.4–71.6)**	**84.8 (84.2–85.4)**
Month 7	100.4 (97.0-103.8)	127.7 (124.3–131.1)	71.3 (69.7–73.0)	84.5 (82.9–86.1)
Month 8	97.3 (93.3-104.3)	126.8 (119.1–133.8)	69.0 (66.0–72.0)	85.8 (82.8–88.7)
Month	100.3 (98.7-101.8)	130.4 (128.9–132.0)	71.1 (70.4–71.8)	84.8 (84.1–85.5)
**Fourth Quarter**	**101.7 (100.6-102.9)**	**133.1 (132.0–134.3)**	**71.0 (70.6–71.5)**	**84.1 (83.7–84.6)**
Month 10	100.3 (97.8-102.7)	132.8 (130.4–135.3)	70.5 (69.4–71.6)	84.1 (N/A, N/A)^a^
Month 11	103.6 (99.0-108.2)	127.3 (122.6–131.9)	69.7 (66.9–72.6)	83.8 (80.9–86.6)
Month 12	102.2 (100.8-103.5)	133.5 (132.1–134.8)	71.3 (70.8–71.8)	83.8 (83.3–84.3)
**Year 2**				
**First Quarter**	**103.0 (101.6-104.4)**	**133.7 (132.3–135.1)**	**69.9 (69.2–70.6)**	**85.0 (84.3–85.7)**
Month 13	102.2 (100.0-104.5)	131.6 (129.4–133.9)	69.3 (68.1–70.5)	85.3 (84.0–86.5)
Month 14	96.7 (91.5-101.8)	137.0 (131.9–142.2)	68.9 (66.7–71.1)	86.5 (84.3–88.7)
Month 15	105.5 (103.7-107.2)	133.7 (132.0–135.5)	70.5 (69.6–71.3)	84.5 (83.7–85.4)
**Second Quarter**	**102.7 (101.3-104.0)**	**134.0 (132.7–135.4)**	**69.9 (69.3–70.5)**	**82.7 (82.2–83.4)**
Month 16	107.3 (104.9-109.7)	131.4 (129.0–133.8)	69.3 (67.9–71.0)	83.8 (82.4–85.1)
Month 17	102.2 (97.4-107.1)	130.0 (N/A, N/A)^a^	68.7 (66.0–71.4)	86.8 (84.1–89.5)
Month 18	101.6 (99.9-103.3)	134.6 (132.9–136.3)	68.3 (67.7–70.8)	83.1 (82.5–83.8)
**Third Quarter**	**104.8 (103.0106.4)**	**134.9 (133.1–136.8)**	**70.2 (69.4–71.0)**	**81.9 (81.6–82.8)**
Month 19	105.3 (103.1-107.5)	134.6 (132.4–136.8)	70.3 (69.3–71.3)	83.3 (82.3–84.3)
Month 20	103.2 (98.9-107.6)	135.7 (131.4–140.1)	69.0 (67.0–71.0)	84.1 (82.1–86.1)
Month 21	103.7 (97.1-110.4)	136.4 (129.7–143.1)	73.1 (71.6–74.6)	80.1 (78.6–81.6)
**Fourth Quarter**	**105.7 (104.7-106.6)**	**132.1 (131.0–133.3)**	**68.9 (67.4–69.2)**	**83.4 (82.4–84.3)**
Month 22	107.3 (102.6-112.0)	136.8 (132.1–141.5)	70.0 (67.5–72.6)	85.4 (82.8–88.0)
Month 23	103.0 (96.91-09.2)	134.2 (128.1–140.4)	71.3 (68.6–73.9)	83.8 (81.1–86.5)
Month 24	105.9 (104.7-107.1)	131.5 (130.3–132.7)	68.9 (67.4–69.2)	83.4 (82.3–84.3)
**Year 3**				
**First Quarter**	**106.0 (104.6-107.7)**	**132.3 (130.8–133.8)**	**69.0 (67.2–71.8)**	**83.3 (82.1–85.2)**
Month 25	106.0 (104.0-108.0)	132.6 (130.7–134.4)	69.6 (68.6–70.6)	83.9 (82.9–84.9)
Month 26	104.5 (101.3-107.8)	131.9 (128.6–135.2)	71.9 (70.4–73.3)	84.3 (82.9–85.7)
Month 27	110.5 (106.8-114.1)	131.0 (127.3–134.6)	72.3 (70.4–74.1)	82.9 (81.0–84.7)
**Second Quarter**	**106.9 (104.1-109.7)**	**133.5 (130.7–136.2)**	**71.2 (69.8–72.6)**	**84.7 (83.3–86.1)**
Month 28	106.2 (101.8-110.7)	132.8 (128.4–137.3)	71.6 (69.6–73.7)	83.8 (81.8–85.8)
Month 29	102.8 (95.6-110.0)	135.1 (127.8–142.3)	73.6 (71.2–76.1)	85.1 (82.6–87.5)
Month 30	110.2 (106.2-114.1)	133.1 (129.2–137.1)	69.6 (67.0–72.2)	85.1 (82.5–87.7)
**Third Quarter**	**103.9 (100.1-107.6)**	**132.1 (128.3–135.8)**	**72.6 (71.3–74.0)**	**83.0 (81.7–84.4)**
Month 31	100.6 (94.5-106.7)	133.9 (127.8–140.0)	73.0 (71.2–74.9)	82.9 (81.0–84.8)
Month 32	–	–	–	–
Month 33	–	–	–	–
**Fourth Quarter**	**105.8 (102.6-108.9)**	**132.7 (129.5–135.8)**	**72.4 (70.8–73.9)**	**85.3 (83.8–86.8)**
Month 34	–	–	–	–
Month 35	104.9 (99.3-110.5)	132.2 (126.6–137.8)	71.4 (68.6–74.2)	85.5 (82.7–88.3)
Month 36	108.4 (104.5-112.4)	130.5 (126.6–134.4)	71.5 (69.1–74.0)	85.2 (82.8–87.7)

Confidence intervals for non-parametric estimates cannot be produced when *n* <120

## Discussion

We have presented age- and sex-specific reference intervals for hemoglobin and mean corpuscular volume (MCV) in an ethnically diverse community sample of Canadian children 2 weeks to 36 months of age. Considering that our findings showed no difference between females and males, we also provided reference intervals for the combined population, maximizing sample size, and hence increasing accuracy and precision of our estimates. This is particularly important for partitions involving younger children, for whom sample size is often very small. We considered large age categories (3 months, 6 months) as well as smaller categories (monthly) to explore the hemoglobin and MCV trajectory for young children with respect to age.

For hemoglobin, from 2 months to 36 months of age, our results show a reference interval (from lower to upper limit) of approximately 30 g/L and the 90% confidence intervals indicate that there is little difference across age groups (approximately 2 g/L each year) or according to sex. These findings raise the possibility that a single reference interval could be used for young children in this age group. For children in the age group 2 weeks to 3 months, we had an adequate sample (> 20) of children at 2 months of age to assess the reference interval and did not detect a lower hemoglobin level consistent with the physiologic nadir, as described by others [19–21]. However, further studies in this very young age group with more data are needed.

For MCV, our results showed a considerable decrease in the reference interval from 2 months of age (79.1–95.5 fL) to 7 months of age (71.0–84.8 fL). The reference interval was lowest during the second year of life, followed by a slight increase in the last months of the third year of life. After 6 months of age, the monthly differences were 1–2 fL.

Two previous publications used CLSI guidelines to establish hematologic reference intervals for healthy young children from China and Korea [13, 14]. Wang et al. established reference intervals for Chinese children. For infants 4 to 6 months of age (*n* = 66 males and 70 females), the reference interval for hemoglobin was 101–134 g/L and for MCV was 69.5–86.7 fL [13]. For infants > 7 months to 1 year (sample size not shown), the reference interval for hemoglobin was 100–134 g/L and for MCV was 63.7–86.3 fL. The findings in this study of healthy Chinese children were similar to our findings in healthy Canadian children, except that the lower limit of MCV appears lower for Chinese infants > 7 months to 1 year.

Lee et al. established hematologic reference intervals in 534 healthy Korean children at 1 year of age, excluding those with evidence of iron deficiency (low MCV, serum ferritin or transferrin saturation) [14]. The reference interval for hemoglobin was 107–140 g/L and for MCV 72.5–83.5 fL [13]. It is difficult to compare the findings from the study in Korean children with our findings, as we did not exclude children with iron deficiency.

Soldin et al. published hematologic reference intervals using data from a large sample of US children undergoing blood sampling in hospital settings (inpatient, outpatient clinics and emergency department) at Boston Children’s Hospital, Boston, MA and Children’s National Medical Center, Washington DC [6]. More recently, Staffa et al. obtained data from the Boston Children’s Hospital Hematology Laboratory, from children referred from 132 Boston area primary care pediatric practices [22]. Neither study used CLSI guidelines. Instead, due to the indirect sampling design, a modified Hoffman method was applied to derive percentile estimates. This statistical methodology is limited by its graphical nature, with CLSI guidelines describing such methods as tools for rough estimation and many authors proposing more advanced automated methods, including maximum likelihood estimation, be used for indirect estimations. In both studies, for infants 2 to 6 months of age, the lower limit of the reference interval for hemoglobin, was mid-90s g/L, whereas in our sample the lower limit for this age group was higher. The differing reference intervals may be explained by differences in subject selection (healthy children versus children undergoing blood sampling for acute or chronic conditions) and methodologic approaches (CLSI approach versus modified Hoffman approach).

Aldrimer et al. calculated hematology reference intervals in Caucasion Swedish children 6 months and 18 years recruited in child healthcare centers and schools [23]. Age- and sex-specific reference intervals were defined by calculating 2.5th and 97.5th percentiles. To create age partitions, the investigators used ‘qualified guessing’ based on visualization of their data. The youngest age group consisted of 91 children 6 months to 7 years of age. With females and males combined, the reference interval for hemoglobin was 107 to 134 g/L, and for MCV was 71.6 to 85.1 fL. The age range of children studied by Aldrimer et al. differed from that of our study, and this likely explains the difference in the hemoglobin reference interval.

Zierk et al. calculated continuous reference intervals using an indirect approach [7]. A laboratory database of samples from children who were inpatients and outpatients at the University Hospital Erlangen, Germany, a pediatric tertiary care center, was used in this analysis. Continuous reference intervals showed that there was a decline in the concentration of hemoglobin during the first months and years of life followed by a subsequent rise. MCV decreased during infancy and stabilized thereafter. Discrete lower and upper reference limits were not provided.

Adeli et al. established hematologic reference intervals using CLSI guidelines across pediatric, adult and geriatric ages using data from the Canadian Health Measures Survey [2]. However, the pediatric age group only included children 3 years and older and analytes were measured using the Beckman Coulter HmX analyzer. For children 3 to 5 years, the hemoglobin reference interval was 113.5 to 143.1 g/L, and the MCV reference interval was 77.2 to 89.5 fL. These reference intervals for children 3 to 5 years overlap with, and demonstrate a continued increase from, the reference intervals we have established for children under 3 years of age. We have previously shown reference intervals for children up to 10 years of age, but did not examine reference intervals by monthly age partitions [12].

For clinical decision making, reference intervals have inherent limitations, as they provide two limits (a lower and upper limit) in a referent population. Decision limits differ from reference intervals and provide clinicians with one cut-point differentiating individuals with disease/no disease [24]. High quality decision limits are based on clinical outcome studies, which are often not available [25]. Therefore, many decision limits are based on consensus. For young children 6 to 59 months, the World Health Organization defines anemia as a hemoglobin less than 110 g/L [26]. This cut-point is higher than the lower limit of the reference interval; therefore, clinicians may misclassify a child’s anemia status if only using the lower limit of the reference interval. Furthermore, the optimal hemoglobin for this age group remains unclear. In a previous analysis we found that as serum ferritin increased, hemoglobin increased and plateaued at approximately 120 g/L; and that at a cut-point of 110 g/L, serum ferritin may be as low as 2.4 μg/L [27]. More research is needed to determine the best decision limit for hemoglobin.

Strengths of our study include the analysis of data following CLSI guidelines, and that data were obtained from a large sample of healthy, ethnically diverse young children recruited during health supervision visits in primary care. Important limitations include the smaller sample size for infants under 4 months of age, and of the individual ethnicity groups. Therefore, we were unable to provide reference intervals in these groups.

## Conclusion

We have established age- and sex-specific reference intervals for hemoglobin and mean corpuscular volume (MCV) in an ethnically diverse community sample of healthy Canadian children 36 months and younger using the CLSI guidelines. As these reference intervals differ substantially from those for older children and adults, clinical laboratories may consider adopting these reference intervals. Further research is needed to establish reference intervals for these hematologic parameters for very young children under 4 months of age.

## Supplementary Information

**Additional file 1: Table A1.** Sample size, skewness and *p*-values from a normality test for each of the partitions each corresponding to Table 2. **Table A2.** Methods used for estimating the lower and upper limits of the reference intervals provided in Table 2, where P, NP and R represent the parametric, non-parametric and robust methods. The methods are selected according to sample size and distributional investigations provided in Table A1. **Table A3.** Sample size, skewness and *p*-values from a normality test for each of the partitions each corresponding to Table 3. **Table A4.** Methods used for estimating the lower and upper limits of the reference intervals provided in Table 3, where P, NP and R represent the parametric, non-parametric and robust methods. The methods are selected according to sample size and distributional investigations provided in Table A3. **Table A5.** Sample size, skewness and *p*-values from the test of normality corresponding to partitions used in Table 4. **Table A6.** Methods used for estimating the lower and upper limits of the reference intervals provided in Table 4, where P, NP and R represent the parametric, non-parametric and robust methods. The methods are selected according to sample size and distributional investigations provided in Table A5. **Table A7.** Sample size for monthly age partitions for females and males.

## Data Availability

The datasets generated and/or analysed during the current study are not publicly available due privacy but are available from the corresponding author on reasonable request.

## Abbreviations

CLSI: Clinical and Laboratory Standards Institute
CI: Confidence interval
MCV: Mean corpuscular volume
