# Supplementary material for: Reference intervals for hemoglobin and mean corpuscular volume in an ethnically diverse community sample of Canadian children 2 to 36 months
Source: BMC Pediatr. 2021 May 19;21:241. doi: 10.1186/s12887-021-02709-w (PMC8132375; doi:10.1186/s12887-021-02709-w)
Supplement: Supplementary file 1 — Additional file 1: Table A1. Sample size, skewness and p-values from a normality test for each of the partitions each corresponding to Table 2. Table A2. Methods used for estimating the lower and upper limits of the reference intervals provided in Table 2, where P, NP and R represent the parametric, non-parametric and robust methods. The methods are selected according to sample size and distributional investigations provided in Table A1. Table A3. Sample size, skewness and p-values from a normality test for each of the partitions each corresponding to Table 3. Table A4. Methods used for estimating the lower and upper limits of the reference intervals provided in Table 3, where P, NP and R represent the parametric, non-parametric and robust methods. The methods are selected according to sample size and distributional investigations provided in Table A3. Table A5. Sample size, skewness and p-values from the test of normality corresponding to partitions used in Table 4. Table A6. Methods used for estimating the lower and upper limits of the reference intervals provided in Table 4, where P, NP and R represent the parametric, non-parametric and robust methods. The methods are selected according to sample size and distributional investigations provided in Table A5. Table A7. Sample size for monthly age partitions for females and males. [file 12887_2021_2709_MOESM1_ESM.docx]

**Reference intervals for hemoglobin and mean corpuscular volume in an ethnically diverse community sample of Canadian children 2 to 36 months**

Jemila S. Hamid, PhD,^1^ Eshetu G. Atenafu, MSc,^2^ Cornelia M. Borkhoff, PhD,^3,4^

Catherine S. Birken, MD, MSc,^3,4,5^ Jonathon L. Maguire, MD, MSc,^3,4,5,6^

Mary Kathryn Bohn, BSc,^7,8^ Khosrow Adeli, PhD,^7,8^ Mohamed Abdelhaleem, MBBCh, PhD,^8,9^ Patricia C. Parkin, MD ^3,4,5^

**Affiliations**:

^1^Department of Mathematics and Statistics, University of Ottawa, Ottawa, Canada

^2^Biostatistics Department, Princess Margaret Cancer Center, University Health Network, Toronto, Canada

^3^Pediatric Outcomes Research Team (PORT), Division of Pediatric Medicine and Sick Kids Research Institute, Hospital for Sick Children, Toronto, Canada

^4^Institute of Health Policy, Management and Evaluation, University of Toronto, Toronto, Canada

^5^Department of Pediatrics, Faculty of Medicine, University of Toronto, Toronto, Ontario, Canada

^6^Department of Pediatrics, and Li Ka Shing Knowledge Institute, St. Michael's Hospital, Toronto, Canada

^7^CALIPER Program, Pediatric Laboratory Medicine, The Hospital for Sick Children, Toronto, Canada

^8^Department of Laboratory Medicine & Pathobiology, Faculty of Medicine, University of Toronto, Toronto, Canada

^9^Haematopathology, Pediatric Laboratory Medicine, The Hospital for Sick Children, Toronto, Canada

**Address correspondence to:** Patricia C. Parkin, MD, FRCPC, The Hospital for Sick Children Research Institute, Peter Gilgan Centre for Research and Learning, 686 Bay St, Toronto, ON, Canada M5G 0A4. E-mail: [patricia.parkin@sickkids.ca](mailto:patricia.parkin@sickkids.ca). 416-813-7654

**Table A1.** Sample size, skewness and p-values from a normality test for each of the partitions each corresponding to Table 2.

| Analyte |  | Female | | | Male | | |
| --- | --- | --- | --- | --- | --- | --- | --- |
|  | Age Group | N | skewness | p-value | N | skewness | p-value |
| Hemoglobin | **Year 1** | 360 | 0.08583 | 0.0020* | 425 | -0.04602 | 0.0578 |
|  | Month 1 – Month 6 | 62 | -0.00063 | 0.0114* | 82 | 0.14398 | 0.8110 |
|  | Month 7 – Month 12 | 298 | 0.10334 | 0.0173* | 343 | -0.09109 | 0.0765 |
|  | **Year2** | 441 | -0.11555 | 0.0080* | 503 | -0.19019 | 0.0008* |
|  | Month 13 – Month 18 | 248 | -0.13490 | 0.0592 | 292 | -0.22308 | 0.0042* |
|  | Month 19 – Month 24 | 193 | -0.02783 | 0.0595 | 211 | -0.02024 | 0.0204* |
|  | **Year3** | 129 | 0.16516 | 0.3931 | 154 | -0.08373 | 0.1499 |
|  | Month 25 – Month 30 | 101 | 0.15555 | 0.3790 | 113 | -0.15554 | 0.1372 |
|  | Month 31 – Month 36 | 28 | -0.20384 | 0.0710 | 41 | 0.10397 | 0.3190 |
| MCV | **Year 1** | 356 | 0.42749 | 0.0008* | 428 | 0.73618 | <0.0001* |
|  | Month 1 – Month 6 | 60 | 0.44005 | 0.1894 | 82 | 0.79281 | <0.0001* |
|  | Month 7 – Month 12 | 296 | 0.00199 | 0.0114* | 346 | -0.20075 | 0.0775 |
|  | **Year 2** | 435 | -0.36851 | 0.0014* | 504 | -0.44922 | <0.0001* |
|  | Month 13 – Month 18 | 246 | -0.20064 | 0.0173 * | 292 | -0.44827 | <0.0001* |
|  | Month 19 – Month 24 | 189 | -0.52486 | 0.0592 | 212 | -0.45470 | 0.0032* |
|  | **Year 3** | 126 | -0.59940 | 0.0235* | 154 | -0.20786 | 0.1010 |
|  | Month 25 – Month 30 | 100 | -0.50331 | 0.0595 | 112 | -0.22552 | 0.1712 |
|  | Month 31 – Month 36 | 26 | -0.46066 | 0.3790 | 42 | -0.18789 | 0.3318 |

* The distribution of the analyte significantly deviates from the Gaussian distribution

**Table A2.** Methods used for estimating the lower and upper limits of the reference intervals provided in Table 2, where P, NP and R represent the parametric, non-parametric and robust methods. The methods are selected according to sample size and distributional investigations provided in Table A1.

| Analyte |  | Female | | Male | |
| --- | --- | --- | --- | --- | --- |
|  | Age Group | Lower Limit | Upper Limit | Lower Limit | Upper Limit |
| Hemoglobin | **Year 1** | P | P | P | P |
|  | Month 1 – Month 6 | P | P | P | P |
|  | Month 7 – Month 12 | P | P | P | P |
|  | **Year2** | P | P | NP | NP |
|  | Month 13 – Month 18 | R | P | P | NP |
|  | Month 19 – Month 24 | P | P | R | P |
|  | **Year3** | P | P | R | P |
|  | Month 25 – Month 30 | P | P | P | P |
|  | Month 31 – Month 36 | P | P | P | P |
| MCV | **Year 1** | NP | NP | NP | NP |
|  | Month 1 – Month 6 | P | P | NP | NP |
|  | Month 7 – Month 12 | NP | NP | P | NP |
|  | **Year 2** | NP | NP | NP | NP |
|  | Month 13 – Month 18 | P | P | NP | NP |
|  | Month 19 – Month 24 | NP | NP | NP | NP |
|  | **Year 3** | P | NP | P | P |
|  | Month 25 – Month 30 | P | P | P | P |
|  | Month 31 – Month 36 | P | P | P | P |

**Table A3.** Sample size, skewness and p-values from a normality test for each of the partitions each corresponding to Table 3.

| Analytes | Age groups | N | skewness | p-value |
| --- | --- | --- | --- | --- |
| Hemoglobin | **Year 1** | 785 | 0.01422 | 0.0022* |
|  | Month 1 – Month 6 | 144 | 0.08312 | 0.2240 |
|  | Month 7 – Month 9 | 253 | 0.16210 | 0.2163 |
|  | Month 10- Month 12 | 388 | -0.10936 | 0.0036* |
|  | **Year2** | 944 | -0.14866 | <0.0001* |
|  | Month 13 – Month 15 | 263 | -0.22750 | 0.0072* |
|  | Month 16 – Month 18 | 277 | -0.13565 | 0.0945 |
|  | Month 19 – Month 21 | 134 | -0.12328 | 0.0488* |
|  | Month 22 – Month 24 | 270 | 0.02832 | 0.0274* |
|  | **Year3** | 283 | 0.03921 | 0.1170 |
|  | Month 25 – Month 30 | 214 | 0.02972 | 0.1040 |
|  | Month 31 – Month 36 | 69 | 0.07054 | 0.4610 |
| MCV | **Year 1** | 784 | 0.58463 | <0.0001* |
|  | Month 1 – Month 6 | 142 | 0.72318 | 0.0002* |
|  | Month 7 – Month 9 | 248 | 0.04540 | 0.1567 |
|  | Month 10- Month 12 | 394 | -0.36640 | 0.0035* |
|  | **Year 2** | 939 | -0.40668 | <0.0001* |
|  | Month 13 – Month 15 | 260 | -0.43943 | 0.0075* |
|  | Month 16 – Month 18 | 278 | -0.35150 | 0.0404* |
|  | Month 19 – Month 21 | 138 | -0.45101 | 0.0340* |
|  | Month 22 – Month 24 | 263 | -0.45125 | 0.0008* |
|  | **Year 3** | 280 | -0.36025 | 0.0053* |
|  | Month 25 – Month 30 | 212 | -0.32467 | 0.0635* |
|  | Month 31 – Month 36 | 68 | -0.41827 | 0.0620 |

* The distribution of the analyte significantly deviates from the Gaussian distribution

**Table A4.** Methods used for estimating the lower and upper limits of the reference intervals provided in Table 3, where P, NP and R represent the parametric, non-parametric and robust methods. The methods are selected according to sample size and distributional investigations provided in Table A3.

| Analytes | Age group | Lower Limit | Upper Limit |
| --- | --- | --- | --- |
| Hemoglobin | **Year 1** | P | P |
|  | Month 1 – Month 6 | P | R |
|  | Month 7 – Month 9 | P | P |
|  | Month 10- Month 12 | P | P |
|  | **Year2** | P | P |
|  | Month 13 – Month 15 | P | NP |
|  | Month 16 – Month 18 | P | P |
|  | Month 19 – Month 21 | R | P |
|  | Month 22 – Month 24 | P | P |
|  | **Year3** | P | P |
|  | Month 25 – Month 30 | P | R |
|  | Month 31 – Month 36 | P | R |
| MCV | **Year 1** | NP | NP |
|  | Month 1 – Month 6 | P | P |
|  | Month 7 – Month 9 | P | P |
|  | Month 10- Month 12 | NP | P |
|  | **Year 2** | NP | NP |
|  | Month 13 – Month 15 | NP | NP |
|  | Month 16 – Month 18 | NP | NP |
|  | Month 19 – Month 21 | P | NP |
|  | Month 22 – Month 24 | NP | NP |
|  | **Year 3** | NP | NP |
|  | Month 25 – Month 30 | NP | NP |
|  | Month 31 – Month 36 | R | P |

**Table A5.** Sample size, skewness and p-values from the test of normality corresponding to partitions used in Table 4.

| Age group | Hemoglobin | | | MCV | | |
| --- | --- | --- | --- | --- | --- | --- |
|  | N | skewness | p-value | N | skewness | p-value |
| Year 1 | | | | | | |
| **First quarter** | 34 | -0.184 | 0.325 | 34 | 0.069 | 0.3338 |
| Month 1 | 10 | 0.521 | 0.143 | 10 | -0.840 | 0.2609 |
| Month 2 | 22 | 0.122 | 0.420 | 22 | 0.324 | 0.3217 |
| Month 3 | 2 | NA | NA | 2 | NA | NA |
| **Second quarter** | 110 | 0.026 | 0.177 | 111 | -0.096 | 0.5110 |
| Month 4 | 30 | -0.137 | 0.509 | 31 | -0.197 | 0.2416 |
| Month 5 | 8 | 0.320 | 0.633 | 7 | -0.027 | 0.2103 |
| Month 6 | 72 | 0.022 | 0.423 | 73 | -0.143 | 0.7737 |
| **Third quarter** | 249 | 0.037 | 0.348 | 253 | 0.117 | 0.2020 |
| Month 7 | 34 | 0.251 | 0.389 | 34 | 0.457 | 0.1990 |
| Month 8 | 16 | 1.273 | 0.012 | 17 | -0.443 | 0.4035 |
| Month 9 | 199 | -0.062 | 0.388 | 202 | 0.164 | 0.1758 |
| **Fourth quarter** | 391 | -0.064 | 0.008 | 392 | -0.261 | 0.0310 |
| Month 10 | 91 | -0.183 | 0.223 | 90 | -0.548 | 0.0270 |
| Month 11 | 14 | -0.178 | 0.150 | 13 | -0.679 | 0.2339 |
| Month 12 | 286 | -0.029 | 0.013 | 289 | -0.120 | 0.3049 |
| Year 2 | | | | | | |
| **First quarter** | 259 | -0.308 | 0.011 | 261 | -0.340 | 0.0917 |
| Month 13 | 88 | -0.172 | 0.246 | 88 | -0.296 | 0.2688 |
| Month 14 | 32 | -0.444 | 0.819 | 33 | -0.574 | 0.3046 |
| Month 15 | 139 | -0.157 | 0.046 | 140 | -0.274 | 0.1298 |
| **Second quarter** | 275 | -0.106 | 0.147 | 278 | -0.418 | 0.0105 |
| Month 16 | 53 | 0.020 | 0.933 | 56 | -0.342 | 0.0135 |
| Month 17 | 23 | -0.919 | 0.040 | 24 | -0.948 | 0.1106 |
| Month 18 | 199 | 0.004 | 0.162 | 198 | -0.370 | 0.0287 |
| **Third quarter** | 134 | -0.123 | 0.049 | 133 | -0.479 | 0.0186 |
| Month 19 | 92 | -0.148 | 0.200 | 91 | -0.448 | 0.0919 |
| Month 20 | 29 | 0.029 | 0.157 | 30 | -0.506 | 0.2208 |
| Month 21 | 13 | -0.294 | 0.274 | 12 | 0.082 | 0.6553 |
| **Fourth quarter** | 268 | -0.003 | 0.021 | 261 | -0.520 | 0.0004 |
| Month 22 | 21 | 0.001 | 0.635 | 19 | -0.113 | 0.6389 |
| Month 23 | 14 | 0.096 | 0.191 | 12 | 0.100 | 0.2497 |
| Month 24 | 233 | -0.056 | 0.040 | 230 | -0.575 | 0.0001 |
| Year 3 | | | | | | |
| **First quarter** | 163 | -0.171 | 0.084 | 164 | -0.475 | 0.0195 |
| Month 25 | 109 | -0.365 | 0.072 | 108 | -0.410 | 0.0829 |
| Month 26 | 37 | 0.583 | 0.104 | 39 | -0.433 | 0.4575 |
| Month 27 | 17 | -0.593 | 0.113 | 17 | -0.495 | 0.2872 |
| **Second quarter** | 48 | -0.183 | 0.642 | 50 | -0.581 | 0.3260 |
| Month 28 | 19 | -0.126 | 0.076 | 19 | -0.058 | 0.9915 |
| Month 29 | 11 | -0.067 | 0.572 | 12 | -0.108 | 0.9012 |
| Month 30 | 18 | 0.011 | 0.981 | 19 | -0.777 | 0.3265 |
| **Third quarter** | 30 | 0.213 | 0.593 | 30 | -0.506 | 0.1393 |
| Month 31 | 16 | 0.260 | 0.551 | 15 | -0.334 | 0.0630 |
| Month 32 | 7 | 0.410 | 0.607 | 7 | -0.316 | 0.5994 |
| Month 33 | 7 | -0.629 | 0.583 | 8 | -0.556 | 0.3519 |
| **Fourth quarter** | 38 | 0.150 | 0.835 | 38 | -0.539 | 0.1222 |
| Month 34 | 8 | 0.326 | 0.910 | 7 | 0.038 | 0.7025 |
| Month 35 | 13 | 0.047 | 0.896 | 14 | -0.068 | 0.8976 |
| Month 36 | 17 | -0.204 | 0.679 | 17 | -0.509 | 0.0867 |

**Table A6.** Methods used for estimating the lower and upper limits of the reference intervals provided in Table 4, where P, NP and R represent the parametric, non-parametric and robust methods. The methods are selected according to sample size and distributional investigations provided in Table A5.

| Age group | Hemoglobin | | MCV | |
| --- | --- | --- | --- | --- |
|  | Lower | Upper | Lower | Upper |
| Year 1 | | | | |
| First quarter | P | P | P | P |
| Month 1 | - | - | - | - |
| Month 2 | P | P | P | P |
| Month 3 | - | - | - | - |
| Second quarter | P | P | P | P |
| Month 4 | P | P | P | P |
| Month 5 |  |  |  |  |
| Month 6 | P | P | P | P |
| Third quarter | P | P | P | P |
| Month 7 | P | P | P | P |
| Month 8 | R | R | P | P |
| Month 9 | P | P | P | P |
| Fourth quarter | P | P | P | P |
| Month 10 | P | P | P | NP |
| Month 11 | P | P | P | P |
| Month 12 | P | P | P | P |
| Year 2 | | | | |
| First quarter | P | P | P | P |
| Month 13 | P | P | P | P |
| Month 14 | P | P | P | P |
| Month 15 | R | P | P | P |
| Second quarter | P | P | P | NP |
| Month 16 | P | P | R | P |
| Month 17 | P | NP | P | P |
| Month 18 | P | P | NP | P |
| Third quarter | R | P | P | NP |
| Month 19 | P | P | P | P |
| Month 20 | P | P | P | P |
| Month 21 | P | P | P | P |
| Fourth quarter | R | P | NP | NP |
| Month 22 | P | P | P | P |
| Month 23 | P | P | P | P |
| Month 24 | R | P | NP | NP |
| Year 3 | | | | |
| First quarter | R | P | NP | NP |
| Month 25 | R | P | P | P |
| Month 26 | P | P | P | P |
| Month 27 | P | P | P | P |
| Second quarter | P | P | P | P |
| Month 28 | P | P | P | P |
| Month 29 | P | P | P | P |
| Month 30 | P | P | P | P |
| Third quarter | P | P | P | P |
| Month 31 | P | P | P | P |
| Month 32 | - | - | - | - |
| Month 33 | - | - | - | - |
| Fourth quarter | P | P | P | P |
| Month 34 | - | - | - | - |
| Month 35 | P | P | P | P |
| Month 36 | P | P | P | P |

**Table A7.** Sample size for monthly age partitions for females and males

| **Age in months** | **Hemoglobin** | | **MCV** | |
| --- | --- | --- | --- | --- |
|  | **female** | **male** | **female** | **Male** |
| 1 | 5 | 6 | 5 | 6 |
| 2 | 7 | 16 | 7 | 16 |
| 3 | 1 | 1 | 1 | 1 |
| 4 | 12 | 19 | 12 | 19 |
| 5 | 3 | 5 | 3 | 5 |
| 6 | 36 | 40 | 36 | 40 |
| 7 | 23 | 12 | 23 | 12 |
| 8 | 9 | 8 | 9 | 8 |
| 9 | 81 | 132 | 81 | 132 |
| 10 | 48 | 47 | 48 | 47 |
| 11 | 8 | 6 | 8 | 6 |
| 12 | 143 | 159 | 143 | 159 |
| 13 | 43 | 51 | 43 | 51 |
| 14 | 14 | 20 | 14 | 20 |
| 15 | 68 | 79 | 68 | 79 |
| 16 | 23 | 35 | 23 | 35 |
| 17 | 10 | 15 | 10 | 15 |
| 18 | 100 | 108 | 100 | 108 |
| 19 | 49 | 47 | 49 | 47 |
| 20 | 17 | 15 | 17 | 15 |
| 21 | 4 | 10 | 4 | 10 |
| 22 | 13 | 8 | 13 | 8 |
| 23 | 8 | 6 | 8 | 6 |
| 24 | 107 | 133 | 107 | 133 |
| 25 | 51 | 62 | 51 | 62 |
| 26 | 20 | 21 | 20 | 21 |
| 27 | 7 | 11 | 7 | 11 |
| 28 | 11 | 9 | 11 | 9 |
| 29 | 8 | 4 | 8 | 4 |
| 30 | 10 | 10 | 10 | 10 |
| 31 | 5 | 11 | 5 | 11 |
| 32 | 5 | 3 | 5 | 3 |
| 33 | 3 | 5 | 3 | 5 |
| 34 | 3 | 5 | 3 | 5 |
| 35 | 4 | 10 | 4 | 10 |
| 36 | 9 | 9 | 9 | 9 |
